# Omnipresence of Weak Antilocalization (WAL) in Bi_2_Se_3_ Thin Films: A Review on Its Origin

**DOI:** 10.3390/nano11051077

**Published:** 2021-04-22

**Authors:** Rubén Gracia-Abad, Soraya Sangiao, Chiara Bigi, Sandeep Kumar Chaluvadi, Pasquale Orgiani, José María De Teresa

**Affiliations:** 1 Departamento de Física de la Materia Condensada, Universidad de Zaragoza, 50009 Zaragoza, Spain; rbgracia@unizar.es; 2 Laboratorio de Microscopías Avanzadas (LMA), Universidad de Zaragoza, 50018 Zaragoza, Spain; 3 Instituto de Nanociencia y Materiales de Aragón (INMA), CSIC-Universidad de Zaragoza, 50009 Zaragoza, Spain; 4 CNR-IOM, TASC Laboratory in Area Science Park, 34139 Triestre, Italy; cb407@st-andrews.ac.uk (C.B.); chaluvadi@iom.cnr.it (S.K.C.); orgiani@iom.cnr.it (P.O.)

**Keywords:** topological insulator, Berry’s phase, Bi_2_Se_3_ film, weak-antilocalization, magnetotransport

## Abstract

Topological insulators are materials with time-reversal symmetric states of matter in which an insulating bulk is surrounded by protected Dirac-like edge or surface states. Among topological insulators, ${\mathrm{Bi}}_{2}{\mathrm{Se}}_{3}$ has attracted special attention due to its simple surface band structure and its relatively large band gap that should enhance the contribution of its surface to transport, which is usually masked by the appearance of defects. In order to avoid this difficulty, several features characteristic of topological insulators in the quantum regime, such as the weak-antilocalization effect, can be explored through magnetotransport experiments carried out on thin films of this material. Here, we review the existing literature on the magnetotransport properties of ${\mathrm{Bi}}_{2}{\mathrm{Se}}_{3}$ thin films, paying thorough attention to the weak-antilocalization effect, which is omnipresent no matter the film quality. We carefully follow the different situations found in reported experiments, from the most ideal situations, with a strong surface contribution, towards more realistic cases where the bulk contribution dominates. We have compared the transport data found in literature to shed light on the intrinsic properties of ${\mathrm{Bi}}_{2}{\mathrm{Se}}_{3}$, finding a clear relationship between the mobility and the phase coherence length of the films that could trigger further experiments on transport in topological systems.

## 1. Introduction

The discovery of the quantum hall (QH) state back in 1980 opened the door to a totally new paradigm in condensed matter science [1,2]. Up to those days, states of matter were classified considering the symmetries they spontaneously break. For instance, crystalline solids break translational symmetry whereas ferromagnets break rotational symmetry. However, the QH state did not have any spontaneously broken symmetry, and hence, it could not be included in that classification. Instead, its novel characteristics were related to the topology of the band structure.

**Figure 1:**
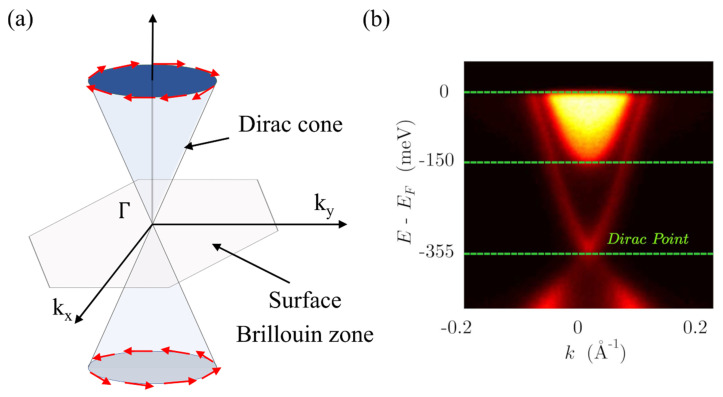
(**a**) Scheme of a Dirac cone at the surface of a Topological Insulator (TI) showing the spin-momentum locking (spin orientations are indicated by red arrows). (**b**) Angle-Resolved Photoemission Spectroscopy (ARPES) image of the ${\mathrm{Bi}}_{2}{\mathrm{Se}}_{3}$ band structure showing the Dirac cone at the center of the Brillouin zone. Reprinted with permission from Reference [7]. Copyright 2010 American Physical Society.

In 2006, a new topological class known as quantum spin hall (QSH) state or topological insulators (TIs) emerged [3,4,5]. In these materials, a strong spin–orbit interaction causes band inversion, creating a non-trivial topology of the bands, which, along with the presence of time reversal symmetry (TRS), produces robust metallic states at the surfaces, whereas the bulk remains insulating. These surface states exhibit a Dirac-like dispersion relation (Figure 1a) with spin momentum locking, preventing the carriers from suffering backscattering against non-magnetic impurities. All these characteristics make them ideal candidates for the observation of exotic phenomena. For example, systems composed of a TI and a superconductor are predicted to host Majorana states, which could be implemented in the development of fault-tolerant quantum computation [5]. In addition, the spin texture of surface carriers has attracted much attention in the field of spintronics [6].

TIs were first experimentally realized in two-dimensional HgTe/CdTe quantum wells [8], and then, in three dimensional systems in the alloy ${\mathrm{Bi}}_{x}{\mathrm{Sb}}_{1-x}$ [9], where the topological nature was demonstrated by imaging its band structure by angle resolved photoemission spectroscopy (ARPES). Soon after that, the discovery of the topological phase in crystals of the stoichiometric materials ${\mathrm{Bi}}_{2}{\mathrm{Se}}_{3},{\;\mathrm{Bi}}_{2}{\mathrm{Te}}_{3},$ and ${\mathrm{Sb}}_{2}{\mathrm{Te}}_{3}$ [10,11] laid the foundations for plenty of new theoretical and experimental works in this field. Many of them have focused their attention on the growth and electronic characterization of TI thin films [12,13,14]. However, even though the topological surface states have been demonstrated systematically through spectroscopic techniques (Figure 1b), their investigation through transport measurements has become very challenging due to the significant contribution from bulk carriers.

**Figure 2:**
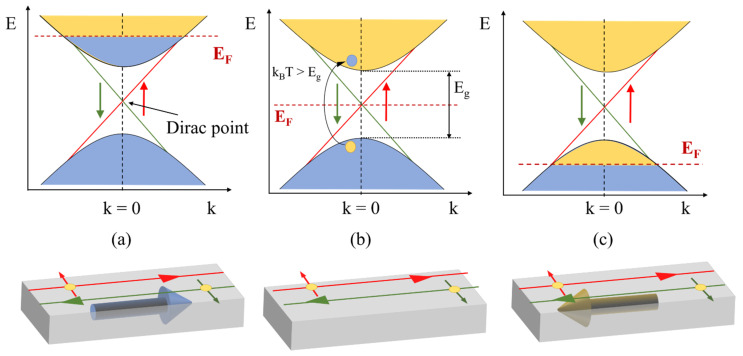
Schemes showing the band structure and the different contributions to transport in TIs: (**a**) n-type conduction with parallel contributions of the surface and the bulk. (**b**) Topological regime with pure surface transport, also indicating the possibility of bulk presence due to thermal activation. (**c**) p-type conduction with surface and bulk contributing. Blue and yellow regions in the bands indicate electron and hole populations, respectively.

A simple treatment of a 3D TI includes the bottom of the bulk conduction band (BCB) and the top of the bulk valence band (BVB), in parallel with the Dirac cones at each of the surfaces. In Figure 2, for the sake of simplicity, we consider the BCB and the BVB in parallel with a single Dirac cone. When the Fermi level lies within the bulk band gap, the topological regime takes place, and the transport occurs entirely through the surfaces (Figure 2b). Unfortunately, naturally formed defects combined with the degradation of the film surface caused by environmental exposure shift the chemical potential towards the BCB (Figure 2a), making the system n-type, and the significant contribution of bulk electrons masks the presence of surface states, hindering their study and manipulation [15,16]. Depletion of bulk carriers has been accomplished by using gating or doping with acceptor ions such as calcium obtaining p-type conduction (Figure 2c) [17,18], but complete elimination of the contribution of the bulk carriers is not always feasible, and is still a matter of research. In the case of narrow-gapped TIs, more difficulties come up even when the topological regime is reached, due to the thermal activation of bulk carriers when the thermal energy, $k_{B}T$, $k_{B}$ being the Boltzmann’s constant and T the temperature, is significantly higher than the band-gap energy $E_{g}$ (Figure 2b).

Over the past decade, new groups of materials have broadened the topological classification beyond TIs. Among these groups we find topological crystalline insulators (TCI), in which surface states are protected by spatial symmetries instead of TRS [19]. An important example is the semiconductor SnTe, predicted first theoretically [20], and then experimentally confirmed [21]. In this material, the metallic surface states are topologically protected by reflection symmetry of the crystal with respect to the {110} mirror plane. The states at the (001) surface present exotic phenomena, such as a Lifshitz transition as the Fermi level passes through the Dirac point. However, the properties of the surface states are tough to observe since it is highly p-doped due to Sn vacancies. Other TCIs with similar properties but with a more accessible topological regime are the alloys ${\mathrm{Pb}}_{x}{\mathrm{Sn}}_{1-x}\mathrm{Te}$ [22], or ${\mathrm{Pb}}_{x}{\mathrm{Sn}}_{1-x}\mathrm{Se}$ [23], in which the Fermi level can be modified more easily. Another interesting group is that formed by topological Kondo insulators (TKI), in which topology and strongly correlated physics come together for the first time. In these systems, protected surface states live in the Kondo gap rather than in the Bloch gap [24]. One paradigmatic case is ${\mathrm{SmB}}_{6}$, where, at low temperatures, the strong interaction of the localized 4f states and the dispersing 5d states of Sm, gives rise to a few meV Kondo gap with the Fermi level right in the middle. Due to this gap, ${\mathrm{SmB}}_{6}$ behaves as an insulator at low temperatures, but unlike ordinary insulators, its resistivity saturates below 4 K. This residual resistivity is attributed to the topological surface states that dominate in that regime. Surface states in ${\mathrm{SmB}}_{6}$ have shown spin polarization, demonstrating their topological nature [25]. Beyond insulators, Dirac/Weyl semimetals have attracted much attention in the last few years. They can be viewed as a 3D generalization of graphene with the conduction and valence bands in the bulk touching each other at some isolated points of momentum space called Weyl nodes [26]. These nodes come in pairs and behave as topologically protected monopoles, each of them carrying opposite monopole charge N. Apart from the so-called single-Weyl semimetals with N=±1, double-Weyl semimetals with N=±2 have also been predicted. The non-trivial characteristics of Weyl/Dirac semimetals allows for the formation of Fermi arcs at the surfaces connecting the projection of bulk Weyl nodes in the surface Brillouin zone. These Fermi arcs were first mapped by ARPES in TaAs [27]. Another very promising group is that of topological superconductors (TS), formed by superconductors with a non-trivial topology of the bands. TSs present unconventional superconducting effects and are expected to host Majorana modes protected by particle–hole symmetry [5]. However, no pure TS has been discovered yet. Instead, these properties can be reached in some modified systems, such as strong-spin orbit materials like InAs in combination with a superconductor [28], or in Cu-doped TI ${\mathrm{Bi}}_{2}{\mathrm{Se}}_{3}$ [29]. In both cases, signs of Majorana modes have been observed as a zero-bias conductance peak in STM studies, demonstrating to be excellent platforms for this new physics. Considering all these new topological classes and their peculiar properties, it can be concluded that topology has significantly enriched the condensed matter physics field and has brought about a new era, with many technological possibilities in the coming future.

**Figure 3:**
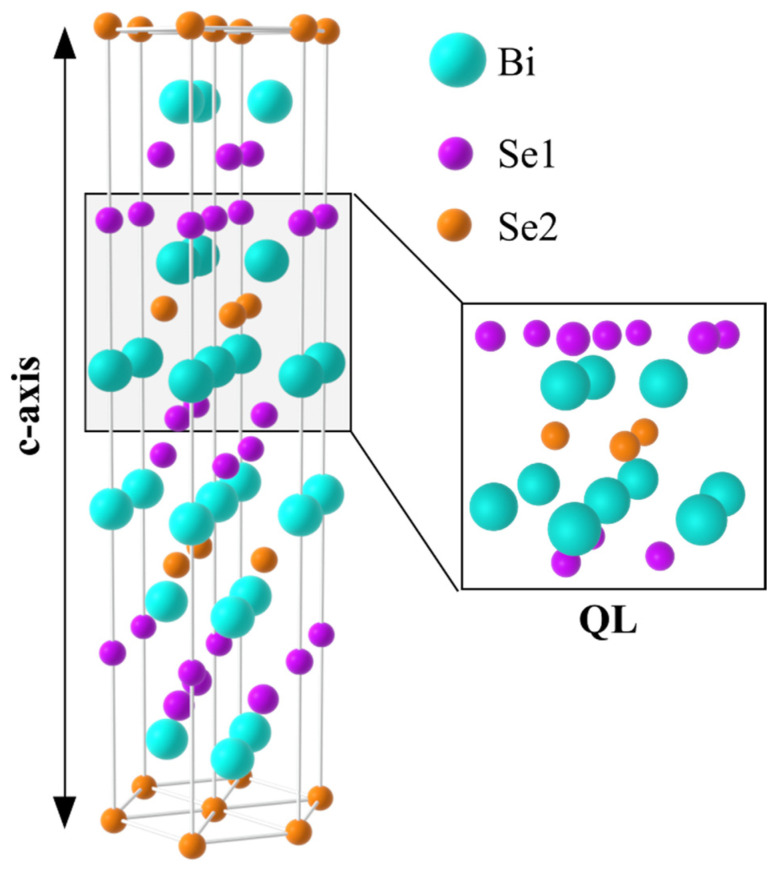
Schematic representation of the unit cell of rhombohedral ${\mathrm{Bi}}_{2}{\mathrm{Se}}_{3}\;$(blue: Bi, violet: Se1, orange: Se2) showing the Quintuple Layer (QL) arrangement and the c-axis direction.

Hereafter, we focus our attention on 3D TI ${\mathrm{Bi}}_{2}{\mathrm{Se}}_{3}$ thin films, which represent a fantastic playground in which to investigate quantum transport phenomena. This material has raised much interest due to its simple surface band structure, consisting of a single Dirac cone (Figure 1b) that allows an easier comparison between experimental and theoretical data, and to its relatively large bulk band gap of 0.3 eV that provides a larger window for the topological regime. It possesses a rhombohedral lattice structure, and it exhibits a periodic arrangement of five atomic layers known as quintuple layer (QL) (1 QL≈1 nm) with a stacking structure Se1–Bi–Se2–Bi–Se1 (Figure 3). Three QLs form the lattice unit cell with a c-axis of 2.86 nm.

Many efforts have been devoted to the development of high-quality ${\mathrm{Bi}}_{2}{\mathrm{Se}}_{3}$ thin films. This is crucial for the incorporation of this material into electronic functional devices in order to carry out the aforementioned applications. Apart from this, the growth of TI thin films presents other advantages: on the one hand, the increase of surface-to-volume ratio enhances the contribution of surface carriers to the transport properties. On the other hand, unlike crystal exfoliation, the growth of films provides a high control on thickness down to few nanometers, which represents an opportunity for the observation and tuning of interesting effects. Several growth techniques such as molecular beam epitaxy (MBE) [30,31,32] or pulsed laser deposition (PLD) [33,34] have proven to be good tools for the study of TI thin films, allowing for the use of different substrates and also the growth of heterostructures. However, the number of defects in the films is still high, and the surface–carrier mobilities are still low, placing TIs far from the technological scenario.

This review is organized as follows: first, an introduction to quantum transport in topological insulators is given, paying special attention to the weak-antilocalization (WAL) effect, which is frequently observed in magnetotransport measurements of ${\mathrm{Bi}}_{2}{\mathrm{Se}}_{3}$ thin films, as well as in other relevant materials with topological properties. Then, we will review some interesting cases reported in the literature in which the connection between the WAL effect and the role of the surface and bulk states will be evidenced. Finally, we will give some interesting insight into the intrinsic properties of ${\mathrm{Bi}}_{2}{\mathrm{Se}}_{3}$ by comparing the data found in the literature.

## 2. Weak-Antilocalization (WAL) Effect

Studying surface transport in TIs is a difficult task due to the presence of bulk carriers that dominate and maintain the system out of the topological regime. The nature of surface states is different from that of bulk states due to their characteristic dispersion relation, which provide them with unique phenomena related to quantum transport and oscillations in electronic properties. These phenomena manifest themselves in transport experiments and allow us to gain insight into the study of these topological states.

### 2.1. Electronic Motion in the Quantum Diffusive Regime

**Figure 4:**
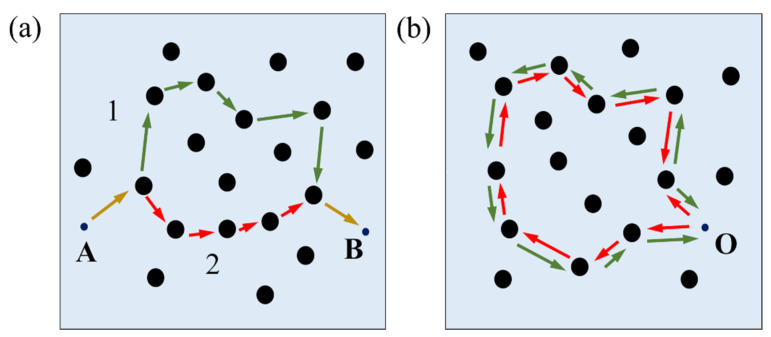
Sketch representing the movement of electrons through scattering centers: (**a**) two possible paths (1 and 2) for an electron going from A to B. (**b**) A loop formed by time reversal partners.

Electronic transport in materials can be classified according to comparisons among characteristic lengths of carriers. As the temperature of a system is decreased, the phase coherence length, $l_{\varphi }$, which defines the average distance an electron can travel until its phase is randomized, can increase and become larger than the elastic mean free path, $l_{e}$. In that case, in a weakly disordered system, the quantum diffusive regime, occurs, and the electrons can move coherently for a relatively long distance while bouncing off the different scattering centers. Taking this into account, the movement of an electron along a path connecting points A and B (Figure 4a), considering paths whose distance is larger than $l_{e}\;$ and shorter than $l_{\varphi }$, can be treated quantum mechanically, and following Feynman formalism, a complex probability amplitude can be attributed to each path that the electron can follow to go from A to B:

(1) $$C_{j}=c_{j}\cdot e^{{i\varphi }_{j}}$$

where j is the label for each possible path, and $\varphi_{j}$ is the phase an electron acquires along that path. The origin of the $\varphi_{j}$ phase is dynamical due to the time variation, as well as geometrical, as demonstrated by Berry [35]. The probability for the electron to go from A to B is determined by the square of the total amplitude:

(2) $$P_{\mathrm{AB}}={\left|\underset{j}{\sum }c_{j}\cdot e^{{i\varphi }_{j}}\right|}^{2}$$

Most of time, phases associated with different paths are randomly distributed and the total probability is averaged out. However, this is different for closed paths (A=B≡O) (Figure 4b) forming a loop. In this situation, each possible path has a time reversal partner with the same probability amplitude, $C_{1,2}=c_{1,2}\cdot e^{{i\varphi }_{1,2}}$, with $c_{1}=c_{2}$ and $\varphi_{1}=\varphi_{2}$, and we have available closed paths as long as these paths are shorter than $l_{\varphi }$. Then, for each loop, the contribution to the current can be expressed as:

(3) PO=|C1+C2|2=|C1|2+|C2|2+2Re(C1*C2)=4|C1|2 

In the classical picture, the interference term goes away, and the total amplitude for the loop is just the sum of the individual probabilities, $2{\left|C_{1}\right|}^{2}$. Considering each loop shorter than $l_{\varphi },$ and applying the same reasoning, it can be observed that quantum interferences have a strong contribution to current and are responsible for an enhancement of the probability of electron backscattering. In practice, this means that the net current is reduced, and, consequently, there is an increase of resistance compared to the classical case. This effect is called weak-localization (WL), and represents a negative correction to conductivity [36].

In materials with strong spin–orbit interaction, the spin orientation is not conserved, which entails an additional phase shift in the wavefunction. In the case of closed paths (Figure 4b), this leads to time reversal partner paths that acquire opposite phases and produce destructive interference. As a result, a decrease in the probability of electron backscattering occurs, resulting in an overall decrease of electrical resistance. This is the weak-antilocalization (WAL) effect, which represents a positive correction to conductivity, as observed in thin films of materials with strong spin–orbit interaction such as Bi [37]. In the surface states of TIs, due to the momentum–spin locking, the spin of the electrons performing a closed path produces a π Berry phase in the electron wavefunction [38]. This gives rise to a purely destructive interference effect between partner paths, producing a strong WAL signature. From now on, we will be focused on this effect, whose detailed analysis provides insight into important transport parameters.

Quantum corrections to resistance produced by WAL disappear in the presence of an external magnetic field that destroys TRS and introduces a phase shift between partner closed paths. This phase shift is loop dependent, since it depends on the magnetic flux piercing through the loop. As a result, the phase shift is randomly distributed among the different loops. As the magnetic field is increased, the strength of the localization effect decreases and, finally, the classical regime is recovered. Quantitatively, the WAL effect can be described by the Hikami–Larkin–Nagaoka (HLN) model [39], which gives the correction to the 2D conductance $∆G_{\mathrm{xx}}$ in the quantum diffusive regime in the presence of a magnetic field B. In the approximation of independent transport channels, and no magnetic scattering, it is expressed as follows:

(4) $$∆G_{\mathrm{xx}}\left(B\right)=-\frac{{\alpha e}^{2}}{2\pi^{2}ћ}\left[\ln\left(\frac{ћ}{4{\mathrm{eBl}}_{\varphi }^{2}}\right)-\psi \left(\frac{1}{2}+\frac{ћ}{4{\mathrm{eBl}}_{\varphi }^{2}}\right)\right]\;$$

where e is the electron charge, ћ is the reduced Planck’s constant, ψ is the digamma function, and α is a parameter representing the nature of the spin–orbit effect in the system, which takes the value of −1/2 for each channel contributing to the WAL effect. In an ideal situation, one expects to find α=−1 corresponding to two independent surfaces, but α=−1/2 is commonly found. In samples with a relevant role of the surface, it can be attributed to an indirect coupling between the top and bottom surfaces through the metallic bulk. In fact, decoupling of the surfaces has been achieved by gating and doping studies, transitioning from α=−1/2 to α=−1 [14,40]. This model provides a methodology for studying WAL experimentally by measuring magnetoresistance in the system and fitting the experimental data to the model, $l_{\varphi }$ and α being the free parameters. On the one hand, the magnitude of $l_{\varphi }$ can be strongly related to the number of defects, and can be used to probe the quality of thin films. Furthermore, $l_{\varphi }$ decreases with increasing temperature due to the thermal activation of phonons. The temperature dependence of $l_{\varphi }\;$ follows a power–law dependence $l_{\varphi }\sim T^{-p/2}$, where p is positive and determined by decoherence mechanisms such as 2D (p=1) and 3D (p=2) electron–electron interaction and electron–phonon interaction (p=3) [41]. On the other hand, α provides knowledge on the channels taking part in the transport. With extra information coming from other magnetotransport measurements, α can be helpful to determine either if the surfaces are contributing to transport or even if the topological regime is taking place. A further study of the dimensionality of the transport can be carried out by studying the WAL effect in tilted magnetic fields. This can distinguish between 2D and 3D character, since 2D transport will depend only on the perpendicular component of the field [42].

**Figure 5:**
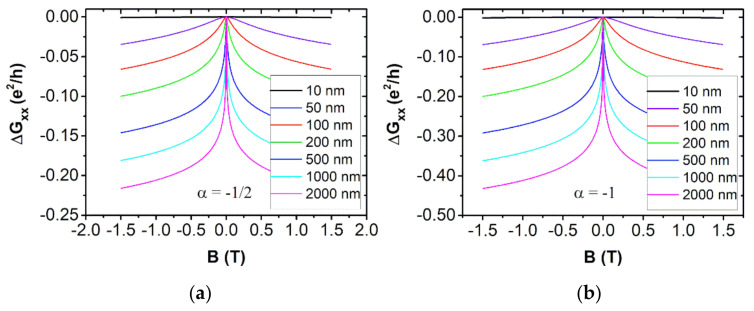
Simulations within the HLN model of the magnetoconductance $∆G_{\mathrm{xx}}\left(B\right)$ for different values of $l_{\varphi }$ at fixed α: (**a**) a single coherent channel α=−1/2. (**b**) Two independent channels α=−1.

In order to obtain a direct interpretation of the model HLN through the experimental magnetotransport data, a detailed simulation of Equation (4) can be carried out by varying the free parameters of the model. By considering that just an integer number of channels can contribute to the coherent transport, α can only take values that are integer multiples of 1/2. Among those cases, α=−1/2, with a single channel, is the most frequently found in ${\mathrm{Bi}}_{2}{\mathrm{Se}}_{3}$ thin films. The minus sign of α appears when the spin–orbit interaction is so strong that the spin number of the carriers is not conserved anymore and, hence, a destructive interference between time reversal closed partner paths occurs (Figure 4b), giving rise to WAL and to a negative correction of the conductance as a function of the magnetic field $∆G_{\mathrm{xx}}\left(B\right)$. On the contrary, a positive value of α appears when the spin–orbit is weak and WL emerges, producing a positive $∆G_{\mathrm{xx}}\left(B\right)$. We are interested in the first case, taking place in TIs. By increasing the phase coherence length $l_{\varphi }$ (Figure 5a) the magnitude of the WAL effect increases, which is translated to more pronounced $∆G_{\mathrm{xx}}\left(B\right)$. This occurs because as the value of $l_{\varphi }$ increases, more and more paths are available to contribute coherently, and then, the weight of quantum corrections like WAL gain weight on the overall transport. For $l_{\varphi }<100\;\mathrm{nm}$, $∆G_{\mathrm{xx}}$ is just a small fraction of the spinless quantum conductance $G_{0}=e^{2}/h=3.35\cdot 10^{-5}\;S$, and one can consider that the WAL is relatively weak in that system. On the other hand, going from α=−1/2 to α=−1, a new coherent channel is introduced, and, in the approximation of independent channels, this results in a doubling of the WAL correction (Figure 5b). Finally, α=0 has been reported few times, and, in that case, the WAL effect is absent. This has been attributed either to a degradation of the topological protection in the ultrathin limit due to direct coupling between the top and bottom surface states [43], or to a motion of carriers in the strong disordered regime ($k_{F}{\;l}_{e}\ll 1$, $k_{F}\;$ being the Fermi wave vector) [44].

### 2.2. WAL in Relevant Materials

The WAL effect is not an exclusive feature of TIs. It is present in materials either with strong spin-orbit interaction or with chiral carriers in the quantum diffusive regime. In the latter case, due to the presence of certain symmetries or interactions, electron states with opposite chirality are not equivalent, which imposes constraints to the type of electron scattering. In topological materials, chiral carriers appear because of the non-trivial topology of the bulk band structure. In TIs, this chirality manifests as spin-momentum locking and is protected by TRS.

Magnetrotransport measurements have shown corrections to magnetoresistance coming from WAL due to spin-momentum locking in TCI SnTe thin films [45]. In this material, four Dirac cones are found at each surface and then, intrasurface and intersurface scattering between the cones can appear. Determining the value of α can provide very useful information about the number of cones taking part in transport and, by changing the Fermi level, allows one to find out how these Dirac cones interact with each other. For instance, it is observed how intersurface coupling is suppressed when the Fermi level lies in the bulk band gap while intrasurface scattering is still present. On the other hand, Dirac/Weyl semimetals can also support corrections to the transport in the quantum diffusive regime. It is originated from the opposite chirality associated to each Weyl node forming the pair due to their opposite monopole charge [46]. The nature of the correction is directly connected to the Berry phase acquired by the electrons performing paths enclosing a Weyl node, and this phase is related to the monopole charge N through πN. In the case of single Weyl semimetal, N=±1, and the Berry phase is equal to π, giving rise to a destructive interference, i.e., the WAL effect and positive magnetoresistance, whereas in the double Weyl semimetals, N=±2  and the Berry phase equals 2π, producing a constructive interference, i.e., the WL effect and negative magnetoresistance. On the other hand, the presence of scattering mechanisms connecting the pair of nodes contributes to localizing carriers and turning WAL into WL [47]. WAL has been confirmed experimentally in topological semimetals such as ${\mathrm{Cd}}_{3}{\mathrm{As}}_{2}$ [48], ${\mathrm{Na}}_{3}\mathrm{Bi}$ [49], or TaAs [50].

These few examples reveal the narrow connection between the quantum corrections to transport in topological systems and their exotic properties, making the study of coherent transport a crucial tool for the complete understanding of these materials, as will also be shown in the particular case of ${\mathrm{Bi}}_{2}{\mathrm{Se}}_{3}$.

## 3. WAL in Bi_2_Se_3_ Thin Films

${\mathrm{Bi}}_{2}{\mathrm{Se}}_{3}\;$ thin films represent an excellent platform for the observation of the WAL effect. In ${\mathrm{Bi}}_{2}{\mathrm{Se}}_{3}$, the spin–orbit interaction is strong and, in the quantum diffusive regime, a positive magnetoresistance is found. In addition, the reduced dimensionality of thin films confines the motion of carriers in a plane, increasing the probability of electrons performing closed paths and then enhancing the WAL effect. The analysis of the WAL effect serves as a perfect complement for the study of electronic transport.

### 3.1. Growth Methods

The incorporation of TI thin films in electronic devices requires high mobility of non-trivial surface carriers and low bulk carrier density. Defects in the lattice structure contribute to transport as dopants. This is the case of ${\mathrm{Bi}}_{2}{\mathrm{Se}}_{3}$, in which n-type selenium vacancies populate the conduction band and make the material metallic. Furthermore, even though surface states in TIs are robust, defects in the surface reduce their mobility and, hence, their contribution to transport. For all this, it is important to develop high-quality thin films to fully exploit the potential of TIs in technological applications. From this point of view, MBE has become the most powerful technique in the epitaxial growth of TIs and other 2D materials, followed by PLD.

In ${\mathrm{Bi}}_{2}{\mathrm{Se}}_{3}$ films grown by MBE, mobilities in the range of $500–1000{\mathrm{cm}}^{2}/(V\cdot s)$ and sheet carrier $n_{2D}$ densities in the order of $10^{13}{\;\mathrm{cm}}^{-2}$ (typical densities at the topological regime are in the order of $10^{12}{\;\mathrm{cm}}^{-2}$) are usually reported [31,51]. Its low growth rate (as low as 0.2−0.3 QLs/min) provides an accurate control on thickness, the growth of films as thin as 2 QLs being common, allowing one to explore exotic phenomena in the ultrathin regime [13,43]. Substrate temperature turned out to be a key parameter in the improvement of films grown by MBE. The use of a two-step growth method, where a few QLs are first deposited at lower temperatures, and then the rest of the film grown under standard conditions, has produced the best films [30,52]. On the other hand, even though PLD is faster than MBE, adatoms arriving onto the substrate have less time to migrate and rearrange and, hence, low defect samples are generally difficult to obtain. Typically, sheet carrier densities in the order of $10^{14}{\;\mathrm{cm}}^{-2}$ (one order of magnitude higher than in MBE-grown samples, indicating more defects) and mobilities in the range of $10-100\;{\mathrm{cm}}^{2}/(V\cdot s)$ are measured [34,53]. Under certain growth conditions, films nearly as good as the ones obtained in MBE with high mobilities close to $500\;{\mathrm{cm}}^{2}/(V\cdot s)$ and low carrier densities have been also reported [54]. Other reported growth methods are chemical vapor deposition (CVD) and magnetron sputtering (MS). CVD has provided good thin films with mobilities around $900{\;\mathrm{cm}}^{2}/(V\cdot s)\;$ and carrier densities close to the topological regime [55,56], whereas MS films reported show low mobilities in the order of $10{\;\mathrm{cm}}^{2}/(V\cdot s)$ and sheet carrier densities in the range of $8–30\cdot 10^{12}{\;\mathrm{cm}}^{-2}$ [57]. 

${\mathrm{Bi}}_{2}{\mathrm{Se}}_{3}$ TI has been grown on a wide variety of substrates, with c-plane sapphire $\left({\mathrm{Al}}_{2}O_{3}\right)$ being the most common. It has the same in-plane lattice structure as ${\mathrm{Bi}}_{2}{\mathrm{Se}}_{3}$, even though there is a significant lattice mismatch (~15%) that can lead to several types of structural defects such as twin defects, antiphase domain, or mosaicity-twist. Apart from sharing the same in-plane structure, sapphire possesses many advantages that make it interesting for ${\mathrm{Bi}}_{2}{\mathrm{Se}}_{3}$ growth: it is low cost, it possesses surface quality, and it is chemically inert. For specific applications, other substrates can be used instead of sapphire. For example, the dielectric properties of ${\mathrm{SrTiO}}_{3}\;\left(111\right)$ or $\mathrm{Si}/{\mathrm{SiO}}_{2}$ make them ideal for gating purposes [44,54]. In ${\mathrm{Bi}}_{2}{\mathrm{Se}}_{3}$ films grown on ${\mathrm{SrTiO}}_{3}\;\left(111\right),$ ambipolar transport has been accomplished several times [12,32]. Transport studies of ${\mathrm{Bi}}_{2}{\mathrm{Se}}_{3}$ thin films grown on GaAs (111) [58], Si (111) [59], ${\mathrm{SiO}}_{2}/\mathrm{graphene}$ [60], and CdS [61] have also been reported, but most of them require surface preparation procedures such as chemical etching, ion bombardment, or temperature annealing in order to obtain atomically flat and clean substrates, which still places sapphire in a favorable position. It is important to notice that compatibility with silicon makes ${\mathrm{Bi}}_{2}{\mathrm{Se}}_{3}$ suitable for the electronic industry.

The influence of substrate choice on the transport properties of thin films has not been clearly demonstrated. In principle, the fact that interactions between QLs are governed by Van der Waals forces should relax the influence of substrate over film properties, even more as film thickness increases. However, ultrahigh mobilities up to $4000\;{\mathrm{cm}}^{2}/(V\cdot s)$ and up to $3500\;{\mathrm{cm}}^{2}/(V\cdot s)\;$ have been found in ${\mathrm{Bi}}_{2}{\mathrm{Se}}_{3}$ grown on CdS [62] and InP (111) [63], respectively. These values are several times higher than values found on standard substrates such as sapphire or ${\mathrm{SrTiO}}_{3}\;\left(111\right)$, highlighting that substrate choice might have consequences on transport properties.

### 3.2. Magnetotransport Properties and WAL Effect in Bi_2_Se_3_ Thin Films

ARPES characterization of ${\mathrm{Bi}}_{2}{\mathrm{Se}}_{3}$ successfully demonstrates the existence of a topological Dirac cone at the surface. Nevertheless, in transport experiments, these topological states are usually obscured by a bulk Fermi surface, which dominates in many occasions. In order to overcome this difficulty, magnetotransport measurements can provide useful information. Both channels, from bulk and surfaces, behave differently, and the application of strong magnetic fields and low temperatures can help to discern their respective contributions.

**Figure 6:**
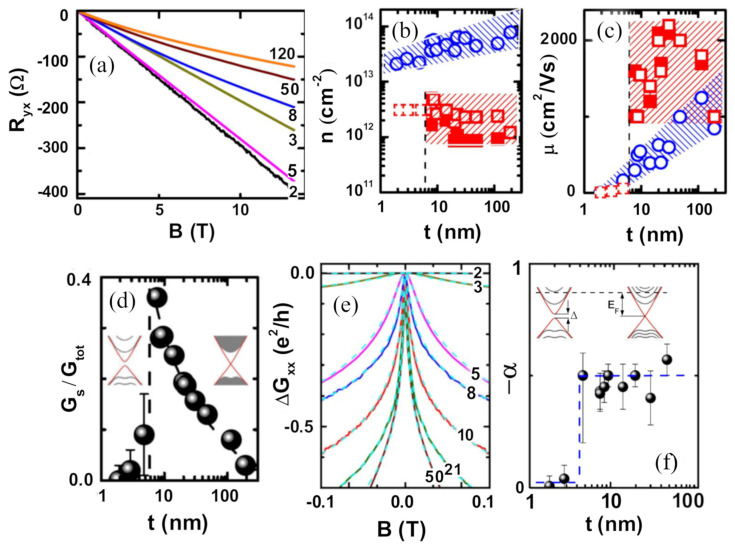
(**a**) Hall resistance against magnetic field for ${\mathrm{Bi}}_{2}{\mathrm{Se}}_{3}$ films with different thicknesses (indicated in QLs on the right part of the plot) showing the non-linear dependence. (**b**) Carrier densities obtained from the SdH oscillations against thickness. (**c**) Mobilities obtained from the two-band analysis against thickness. In (**b**) and (**c**), the blue circles represent the bulk data $\left(n_{b},{\;\mu }_{b}\right)$ and the red squares represent the surface data $\left(n_{s},{\;\mu }_{s}\right)$, where empty and filled squares represent the two different surfaces. (**d**) $G_{s}/G_{\mathrm{tot}}$ against film thickness. (**e**) 2D Magnetoconductance for different film thicknesses. (**f**) Value of α against film thickness. Inset in (**e**) shows schematic energy bands above and below the critical thickness. Data were taken at T=1.6 K. Reprinted with permission from Reference [13]. Copyright 2012 American Physical Society.

In samples with a low density of defects and high mobility, the Fermi level is still above the bottom of the conduction band, but the surface has a relevant role against the bulk, allowing one to separate both contributions by fitting the experimental data to a multiple band model. As long as the two surfaces have comparable mobilities and there are no impurity bands, a two-band model is enough to describe the data, considering one band for the bulk states and another one for the surface states. This parallel contribution of two channels is usually reflected on a non-linear magnetic dependence of the Hall resistance $R_{\mathrm{xy}}$. A detailed study of these contributions is given in [13]. The authors grew ${\mathrm{Bi}}_{2}{\mathrm{Se}}_{3}$ thin films on sapphire by MBE and could observe the evolution of the surface and bulk transport parameters covering a wide range of thicknesses t from 2 QLs  to 200 QLs. $R_{\mathrm{xy}}$ showed deviation from linearity for t>5 QLs (Figure 6a). This feature in the Hall resistance was accompanied by the presence of 2D Shubnikov-de Haas (SdH) oscillations that supported the presence of the surface states and provided constraint parameters for the model. The fitting of the $R_{\mathrm{xy}}$ data yields a contribution of the surface to the total transport $G_{s}/G_{\mathrm{tot}}$ that increases with decreasing t (Figure 6d), reaching values up to ≈0.35, indicating that the bulk still dominates but the surface has a comparable role as the surface-to-volume ratio is increased. The evolution of the sheet carrier density and the mobility of bulk $\left(n_{b},{\;\mu }_{b}\right)$ and surface $\left(n_{s},{\;\mu }_{s}\right)$ carriers with t is shown in Figure 6b,c. Unlike the parameters of the bulk carriers, the parameters of the surface carriers resulted in being almost thickness-independent. Furthermore, even though transport is always dominated by the bulk, the mobility of surface carriers reaches values in the range of $1000–2000\;{\mathrm{cm}}^{2}/(V\cdot s)$, whereas the mobility of the bulk states remains below $1000\;{\mathrm{cm}}^{2}/(V\cdot s)$ for most of the studied thicknesses, implying that the dominance of the bulk states comes from the values of the carrier density $n_{b}$, one order of magnitude higher than $n_{s}$.

At low magnetic fields and low temperatures, the WAL effect is present, and it manifests itself as a negative magnetoconductance (Figure 6e). By using the HLN model, the values of $l_{\varphi }$ and α can be extracted for the different values of $t.{\;l}_{\varphi }$ increases with increasing thickness, reaching values up to  905 nm (see Supporting Information of [13]). This indicates an improvement in the quality of the films for higher thicknesses. The value of α is plotted in Figure 6f against t. For  t>5 QLs, α≈−1/2, but below 5 QLs, it drops abruptly to 0. This is ascribed to a gap opening at the Dirac point below that thickness. This phenomenon has been previously reported in ARPES studies [43] and it is attributed to a direct coupling between the top and the bottom surfaces below a critical thickness (t≈6 QLs), weakening the topological protection of the system and being translated into a diminishment of surface transport, as can be seen in Figure 6d. 

**Figure 7:**
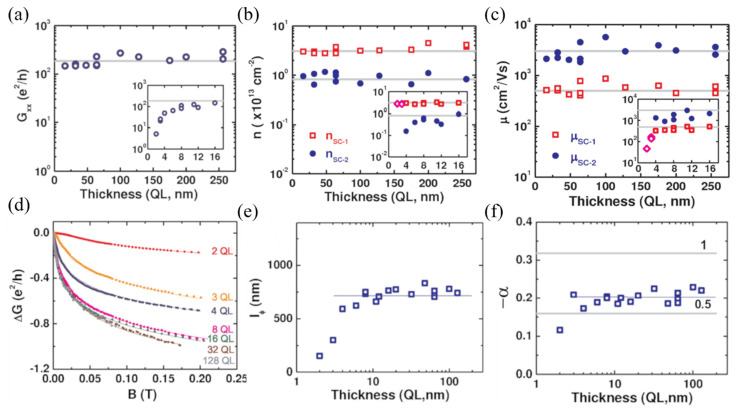
Transport data of ${\mathrm{Bi}}_{2}{\mathrm{Se}}_{3}$ thin films against thickness: (**a**) conductance, (**b**) carrier density, (**c**) mobility. The solid straight lines are guides for the eyes. The subscript ${\mathrm{SC}}_{1}$ corresponds to the topological surface states whereas the subscript ${\mathrm{SC}}_{2}$ corresponds to the 2DEG. (**d**) Magnetoconductance for different thicknesses. (**e**) $l_{\varphi }$ against thickness. (**f**) α against thickness. Insets in (**a**), (**b**), and (**c**) show the data for thinner films. Reprinted with permission from Reference [64]. Copyright 2012 American Physical Society.

In cleaner samples, pure surface transport can be realized. This has been achieved in samples prepared by MBE on sapphire [64], where the Fermi level lies right at the bulk band gap, and the bulk transport remains suppressed in the whole range of studied thicknesses, and, hence, the transport properties are thickness-independent down to the ultra-thin regime (t>8 QLs) where intersurface effects take place. This can be seen in the conductance G data (Figure 7a). However, topological surface states appear to be contributing in parallel to a two dimensional electron gas (2DEG), located right below the Fermi level, arising at the surfaces as a consequence of downward band bending due to environmental exposure [65]. Applying a two-band model, the sheet carrier density and mobility of the topological states $n_{\mathrm{SC}-1}{\;\mathrm{and}\;\mu }_{\mathrm{SC}-1}$, and of the 2DEG $n_{\mathrm{SC}-2},{\;\mathrm{and}\;\mu }_{\mathrm{SC}-2},\;$ can be analyzed (Figure 7b,c). The 2DEG presents a lower sheet carrier density $\left(\sim 8\cdot 10^{12}{\;\mathrm{cm}}^{-2}\right)$ and a higher mobility $\left(3000\;{\mathrm{cm}}^{2}/(V\cdot s\right))$ than the topological surface states $\left(\sim 3\cdot 10^{13}{\;\mathrm{cm}}^{-2},\;500{\;\mathrm{cm}}^{2}/(V\cdot s\right))$. The behavior of the two channels stays thickness-independent for t>8 QLs,  but is qualitatively different below this value. Whereas $n_{\mathrm{SC}-1}$ remains constant, a decrease in $n_{\mathrm{SC}-2}$ is observed (inset in Figure 7b). This is attributed to the fact that this range of thicknesses is comparable to the size of the 2DEG, modifying their energy levels and their density of states due to quantum confinement. On the contrary, $\mu_{\mathrm{SC}-1}$ decreases in the ultra-thin regime (inset in Figure 7c), whereas $\mu_{\mathrm{SC}-2}$ remains constant, indicating that the topological transport is degraded in this regime due to the higher number of defects at the interfaces and to the possible gap opening. SdH effect is observed in the magnetoresistance and its analysis reveals that it arises from the 2DEG, as expected due to its significant higher mobility compared to that of the topological surface states.

The coherent transport also shows thickness-independent behavior. An increase in the magnitude of the WAL effect is commonly observed as the film thickness is decreased due to a confinement of the movement of electrons that enhances their probability for performing closed paths. However, this is not occurring for samples above t=8 QLs (Figure 7d). The value of $l_{\varphi }$ remains close to 750 nm (Figure 7e), and α equals a value between −1 and −1/2 in this range of thicknesses (Figure 7f). One would expect α to be −1 for two decoupled surfaces, or −1/2 for two coupled surfaces. The found value is a combination of both situations and is probably explained by the difficult interplay between the topological surface states and the 2DEG. The thickness-independent $l_{\varphi }$ is a clear indication that the WAL effect originates completely at the surfaces, unlike in [13], where a clear thickness-dependence is observed. Below 8 QLs, both, $l_{\varphi }$ and α decrease with decreasing thickness. This is related to the direct coupling of the surfaces that weakens the topological protection, as indicated in [13].

**Figure 8:**
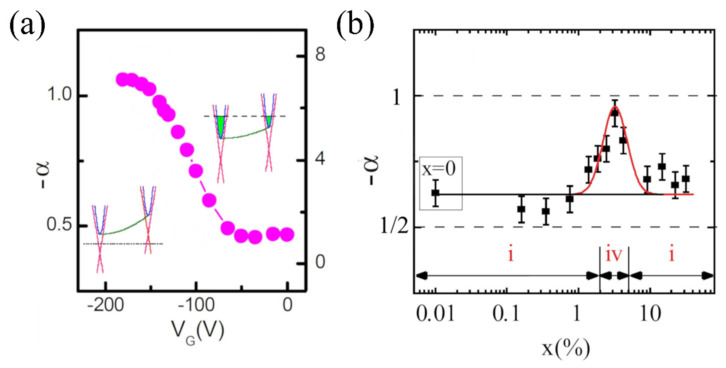
Modulation of α: (**a**) effect of negative gating voltage $V_{G}\;\mathrm{on}\;\alpha$. The insets on the left and right are band diagrams showing the Fermi level modulation relative to the bands. (**b**) Effect of Cu doping (x represent the Cu content) on α. The numbers i and ii correspond to the coupled and decoupled case, respectively. Reprinted with permission from references [32,40]. Copyright 2011 and 2014 American Physical Society.

These results provide insight on the differences in the quantum transport in samples with pure surface transport against samples with bulk contribution. Pure topological transport of ${\mathrm{Bi}}_{2}{\mathrm{Se}}_{3}\;$ has turned out to be challenging without the use of gating or doping. The use of these methods allows for the manipulation of the Fermi level, and, consequently, the modulation of α, going from a situation in which the top and bottom surfaces are coupled  (α=−1/2), to a situation in which the Fermi level lies within the bulk bandgap and the surfaces are independent (α=−1). That is the case of the application of negative gate voltages (Figure 8a) [32] or doping with a proper amount of Cu (Figure 8b) [40].

**Figure 9:**
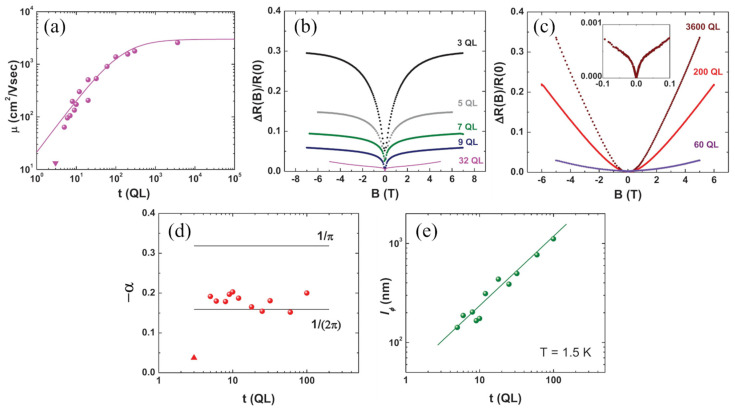
(**a**) Mobility against thickness following the conventional dependence of bulk transport in films, which is indicated by the solid line. (**b**) Magnetoresistance for lower thicknesses. (**c**) Magnetoresistance for high thicknesses showing the $B^{2}\;$ dependence characteristic of bulk dominance. Inset displays the low field region. (**d**) Parameter α against thicknesses. (**e**) $l_{\varphi }\;$ against thickness. Reprinted with permission from Reference [31]. Copyright 2011 American Physical Society.

If the number of defects is higher, the contribution of the surfaces can be totally masked by the bulk, dominating even if the surfaces are active. The dependence of bulk-dominated transport on thickness is properly addressed in [31]. A batch of ${\mathrm{Bi}}_{2}{\mathrm{Se}}_{3}$ thin films with thicknesses ranging from 3 QLs to 3600 QLs are synthesized by MBE on Si (111). The mobility follows a conventional dependence on thickness, increasing in the beginning and saturating for large thicknesses $\left(t\gg l_{e}\right)$ at values around $1300\;{\mathrm{cm}}^{2}/(V\cdot s)$ (Figure 9a), suggesting that the bulk is the main contributor to transport. Furthermore, the sheet carrier density always stays above the characteristic values of the topological regime $\left(\sim 10^{12}{\mathrm{cm}}^{-2}\right)$. For thinner samples, the low magnetic field region of the perpendicular magnetoresistance shows the WAL (Figure 9b) correction, which gets weaker as the thickness increases above few tens of QLs and the $B^{2}$ dependence characteristic of bulk transport becomes more and more evident at higher magnetic fields as the WAL is suppressed (Figure 9c).

The WAL effect can be analyzed following the HLN model, and the obtained coherent parameters are plotted against thickness in Figure 9d,e. The value of α stays close to −1/2 for t>3 QLs, indicating that the WAL is either entirely originated by the bulk, or it is originated by a single channel composed of the two surfaces coupled through the bulk. In the first case, one would expect a linear dependence of $l_{\varphi }$ on thickness. However, a sublinear dependence ($\sim t^{0.7}$) is found, revealing a participation of the surfaces that are coupled through the bulk states.

Finally, in samples with a too high density of defects, the surface mobility is too low, and the transport is totally dominated by the bulk states. We have observed bulk dominated transport on ${\mathrm{Bi}}_{2}{\mathrm{Se}}_{3}$ thin films with different thicknesses going from 15 nm to 100 nm, grown on sapphire by PLD [66] and further fabricated by a standard optical lithography process. The samples were then measured with a physical properties measurement system (PPMS) where high magnetic fields up to 9 T and low temperatures down to 2 K were applied.

**Figure 10:**
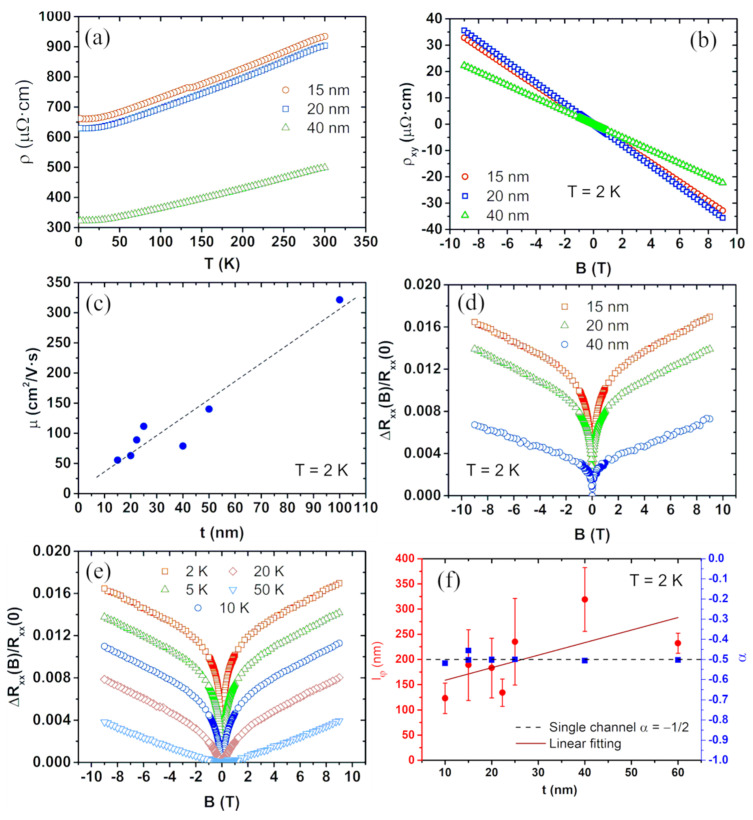
(**a**) Temperature dependence of resistivity for three ${\mathrm{Bi}}_{2}{\mathrm{Se}}_{3}$ films with different thicknesses showing the metallic behaviour (dρ/dT>0). (**b**) Hall resistivity at 2 K against magnetic field for different thicknesses. (**c**) Mobility at 2 K against film thickness. (**d**) Normalized magnetoresistance at 2 K for different thicknesses. (**e**) Normalized magnetoresistance for a 15-nm-thick film at different temperatures. (**f**) Coherent transport parameters $l_{\varphi }$ and α against thickness at 2 K.

The temperature dependence of the resistivity shows a metallic character for all the thicknesses (Figure 10a), indicating that the Fermi level lies somewhere above the bottom of the conduction band. The Hall resistance dependence on the magnetic field was linear with a negative slope in the whole range of thicknesses (Figure 10b), suggesting n-type transport and the dominance of one conduction channel. From the slope of the Hall resistance, the carrier concentration of the films was extracted by applying Drude’s model, being in the order of $10^{20}{\;\mathrm{cm}}^{-3}$, corresponding to a sheet carrier density of $\sim 10^{14}{\;\mathrm{cm}}^{-2}$. This is two orders of magnitude above the topological regime, suggesting that the BCB is highly populated and that the bulk states carry the transport in the samples. The mobilities (Figure 10c), calculated with the carrier densities and the resistivity values, increase with increasing thickness due to the reduction of surface scattering as well as to film quality improvement. The values are found in the range of $50-325{\mathrm{cm}}^{2}/(V\cdot s)$. These relatively low values, together with the high carrier concentrations, indicate a relatively high number of defects in the samples.

The perpendicular magnetoresistance shows WAL correction at low fields, whereas, at higher fields, WAL is suppressed, and the classical behavior is recovered. The WAL effect becomes weaker with increasing thickness (Figure 10d), and for t=100 nm, it is almost absent, even at 2 K. This is explained by the fact that the transport becomes 3D due to the low value of $l_{\varphi }$ in comparison with the thickness. As the film quality improves, $l_{\varphi }$ increases, and it requires higher thicknesses to diminish the WAL effect. The WAL contribution also becomes weaker with increasing temperature (Figure 10e), being almost suppressed above 20 K due to the thermal activation of phonons that act as dephasing scattering centers against carriers.

The coherent transport of samples with t<100 nm was then analyzed through the HLN model, obtaining ${\alpha \;\mathrm{and}\;l}_{\varphi }$ (Figure 10f). α remains close to −1/2 for the whole range of t, whereas $l_{\varphi }$ increases with increasing film thickness indicating an improvement of film quality, as shown in the mobility. The dependence on thickness indicates a contribution dominated by the bulk. We can have either two surfaces coupled through the bulk states, or a metallic bulk completely carrying the coherent transport. Even though the dispersion of the $l_{\varphi }$ values extracted makes it difficult to determine the exact dependence on thickness, a linear relation seems to properly describe the data. Furthermore, considering the high carrier density, as well as the low values of the mobility, we can conclude that α=−1/2 corresponds to the bulk states completely dominating the coherent transport.

As a general picture of the study of magnetotransport and the WAL effect in ${\mathrm{Bi}}_{2}{\mathrm{Se}}_{3}\;$ thin films, a collection of the relevant transport parameters found in the literature are presented in Table 1. Again, the major weight of the bulk states is reflected in the high values of the carrier density, usually one order of magnitude higher than the characteristic of the topological regime $\left(n_{s}\sim 10^{12}{\;\mathrm{cm}}^{-2}\right)$, as well as the low mobilities of some reported cases. These values also reflect how difficult it seems to be to grow ${\mathrm{Bi}}_{2}{\mathrm{Se}}_{3}$ thin films with high mobilities and long coherence lengths. It can also be observed that α is close to −1/2 in the majority of studied cases, revealing the influence of a single transport channel, usually ascribed to the bulk. 

## 4. Remarks and Conclusions

In summary, we have reviewed the study of quantum transport in ${\mathrm{Bi}}_{2}{\mathrm{Se}}_{3}$ thin films, paying attention to the WAL effect, omnipresent in magnetotransport measurements of these systems. We have shown how the parameters that describe this effect evolve from the most ideal case, where the number of defects is low, towards situations where the introduction of defects enhances the contribution of the bulk more and more until it completely dominates the transport. This is reflected in the thickness dependence of transport parameters such as the mobility, μ, or the phase coherence length,$\;l_{\varphi }$, going from an independent behaviour in the first case, to a sublinear dependence, and finally to a linear dependence in the case of a very high number of defects. This is manifested clearly in the analysis of $l_{\varphi }$. The spin-orbit parameter α provides extra information that, together with the magnetotransport data, can help to discern the relevance of the bulk in the transport. In the literature, it is commonly observed that α=−1/2, revealing a partial role of the bulk connecting the two surfaces. Even in [64], we find influence from the bulk in the value of α. This exposes the difficulties in completely avoiding the bulk states in the study of ${\mathrm{Bi}}_{2}{\mathrm{Se}}_{3}$, with gating or doping being necessary to reach the topological regime and take α from −1/2 to −1.

**Figure 11:**
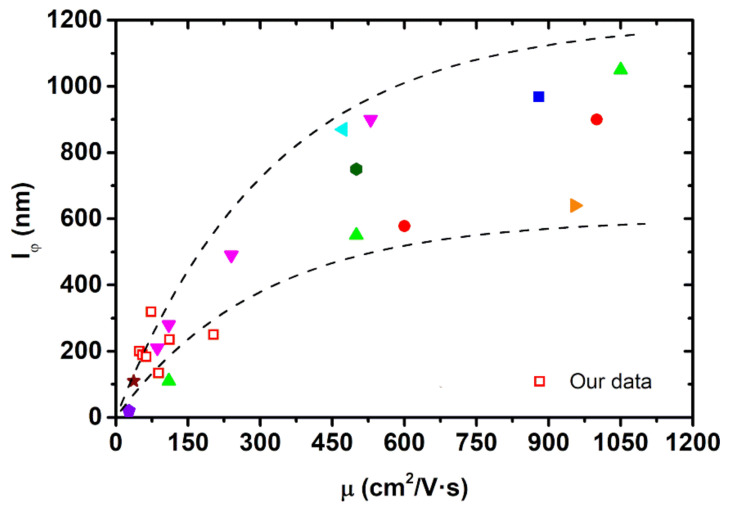
Representation of the phase coherence length $l_{\varphi }$ versus mobility μ values for different films. The dashed black lines are guides for the eyes. Red empty squares correspond to data obtained in our samples. The rest have been taken from literature: Red circles, [13]; blue square, [30]; light green upwards triangle, [31]; cyan left-pointing triangle, [42]; downwards magenta triangles, [54]; right pointing orange triangle, [56]; dark green hexagon, [64]; violet pentagon, [69]; maroon star, [70].

For a deeper analysis of the data reported in Table 1, the phase coherence length ${\;l}_{\varphi }$ data extracted from the literature, together with that of our samples, were plotted against the mobility μ (Figure 11). Comparing them, it looks like, as μ increases, $l_{\varphi }$ saturates, and even though there is a variability in the properties of the films, $l_{\varphi }$ shows a behavior that can be predicted from the mobility of the carriers in the system. A theoretical study of this relation could be used as a means for a deeper understanding of transport in topological systems.

Finally, the data presented in this work demonstrate the potential of ${\mathrm{Bi}}_{2}{\mathrm{Se}}_{3}$ in some of the most researched fields nowadays. The large values of the electron phase coherence length observed for ${\mathrm{Bi}}_{2}{\mathrm{Se}}_{3}$, typically a few hundred nanometers, as shown in Figure 11, indicate that this material is of great interest for applications in spintronics and quantum technologies. In spintronics, a large spin-to-charge conversion has been observed in ${\mathrm{Bi}}_{2}{\mathrm{Se}}_{3}\;$ and ascribed to the presence of surface states and the inverse Rashba-Edelstein effect [71]. In addition, the existence of a large phase coherence length will allow for coherent spin and charge transport along thick ${\mathrm{Bi}}_{2}{\mathrm{Se}}_{3}$ spacers, paving the way for more complex spintronic devices combining ${\mathrm{Bi}}_{2}{\mathrm{Se}}_{3}\;$ and other materials [72]. Regarding quantum technologies, it has been put forward that hybrid ${\mathrm{Bi}}_{2}{\mathrm{Se}}_{3}$-superconductor heterostructures can host Majorana fermions, which is of interest for quantum computing [73]. Thanks to its large phase coherence length, quantum sensors based on ${\mathrm{Bi}}_{2}{\mathrm{Se}}_{3}\;$ can be built too, such as electron interferometers based on the Aharonov–Bohm effect [74] or Josephson junctions where ${\mathrm{Bi}}_{2}{\mathrm{Se}}_{3}$ constitutes the weak link [75].

## Figures and Tables

**Table 1:** Transport parameters of ${\mathrm{Bi}}_{2}{\mathrm{Se}}_{3}$ thin films reported in the literature.

Reference	t (nm)	n_2D_ ${\left(10^{13}\;cm^{-2}\right)}^{\;}$	n $\left(10^{19}\;cm^{-3}\right)$	μ $\left(cm^{2}/\left(Vs\right)\right)$	T (K)	$l_{\varphi }$ (nm)	α
[13]	2–50	-	4	100–1200	1.6	100–1000	−0.5
[14]	20	-	-	-	0.3–10	80–300	−1~−0.5
[30]	≈38		1	390–880	1.8	306–968	−0.52
[31]	1–100	-	0.1–6	70–1150	1.5	150–1000	−0.6~0.5
[32]	5–20	0.8–8.6	-	20–1000	1.2	143–∞	−0.5
[42]	10	-	6	472	0.4–10	150–870	−0.6
[44]	7	1.5	-	-	2.5	55–90	−0.6~−0.2
[51]	1–6	3.5	-	31–350	1.5	75–200	−0.6~−0.3
[54]	6–22	-	3.5–6.5	80–530	2	200–900	−0.55~−0.35
[56]	30	-	1.1	954	2	640	−0.56
[57]	9–54	~100	-	-	2–9	10–159	−1.08~0.16
[64]	10–245	3	-	500	1.5	750	−0.6
[67]	30–300	0.81–3.25	-	-	10	318–879	−0.72~−0.34
[68]	9.8–23	0.3	-	-	0.3–8	300–800	−0.4
[69]	5	-	3	27	2–20	8–20	−0.5
[70]	12	-	4.6	37	1.6–6	65–110	−0.7~−0.6
**Our data**	15–60	7–50	7–50	50–150	2	120–325	~−0.5

## Data Availability

The data presented in this study are available from the corresponding authors upon reasonable request.
